# Parathyroid Hormone Concentrations in Maintenance Hemodialysis: Longitudinal Evaluation of Intact and Biointact Assays

**DOI:** 10.1016/j.xkme.2020.12.015

**Published:** 2021-02-27

**Authors:** Carolin Berner, Rodrig Marculescu, Florian Frommlet, Amelie Kurnikowski, Benjamin Schairer, Christof Aigner, Christian Bieglmayer, Manfred Hecking

**Affiliations:** 1 Division of Nephrology & Dialysis, Department of Medicine III, Medical University of Vienna; 2 Nephrology & Dialysis, 1st Medical Department, Kaiser Franz Josef Hospital Vienna, Medical University of Vienna, Vienna, Austria; 3 Clinical Institute for Medical and Chemical Laboratory Diagnostics, Medical University of Vienna, Vienna, Austria; 4 Center for Medical Statistics, Informatics and Intelligent Systems, Section for Medical Statistics, Medical University of Vienna, Vienna, Austria

**Keywords:** Parathyroid hormone, chronic kidney disease-mineral and bone disorder, immunoassay, follow-up, renal insufficiency, maintenance dialysis

## Abstract

**Rationale & Objective:**

Management of chronic kidney disease mineral and bone disorder requires parathyroid hormone (PTH) concentrations. “Biointact” PTH immunoassays detect “whole” PTH (wPTH), whereas “intact” immunoassays measure PTH plus PTH fragments (iPTH). We aimed to determine whether longitudinal changes in PTH concentrations can be evaluated using biointact and intact immunoassays alike.

**Study Design:**

Open noninterventional longitudinal cohort study.

**Setting & Participants:**

PTH concentrations were measured quarterly up to 5 times in 102 hemodialysis patients.

**Predictors & Tests Compared:**

Age, sex, phosphate levels, and others as clinical predictors for PTH trend. Tests compared were iPTH immunoassays from Siemens and Roche and wPTH immunoassays from Roche and DiaSorin.

**Outcomes:**

PTH concentration trend; regression equations; test bias.

**Analytical Approach:**

Predictive regression-to-the-mean model for PTH slope; Bland-Altman plots, Passing-Bablok regression, and reference change values for test comparisons.

**Results:**

wPTH concentrations were similar with both immunoassays (wPTH-Roche = 11.7 + 0.97 × wPTH-DiaSorin, *r* = 0.99; mean ± 1.96 SD bias, 8.2 ± 43.3 pg/mL [17.5% ± 40.9%], by Bland-Altman plots). iPTH-Siemens concentrations were higher than iPTH-Roche concentrations (iPTH-Siemens = −5.4 + 1.33 × iPTH-Roche, *r* = 0.99; mean ± 1.96 SD bias, 84.0 ± 180.2 pg/mL [21.1% ± 29.8%], by Bland-Altman plots). iPTH-Roche and iPTH-Siemens concentrations were 2- and 2.5-fold higher than wPTH concentrations, respectively. Full agreement among all 4 immunoassays in detecting both significant and insignificant changes in PTH concentrations, upward or downward from one quarter to the next, was reached in 87% of consecutive measurements. In a predictive model, baseline PTH concentrations > 199 pg/mL (wPTH-Roche), 204 pg/mL (wPTH-DiaSorin), 386 pg/mL (iPTH-Roche), and 417 pg/mL (iPTH-Siemens) correctly predicted declining PTH concentration trend in 62% to 68% of patients, but age, sex, hemodialysis vintage, and calcium and phosphate levels were no significant predictors.

**Limitations:**

Limited number of immunoassays, only 59 patients attended all quarterly samplings.

**Conclusions:**

wPTH-Roche and wPTH-DiaSorin concentrations were similar, while iPTH was higher than wPTH concentrations. The iPTH-Siemens immunoassay is either higher calibrated or detects more fragments than iPTH-Roche. However, longitudinal PTH concentration changes largely coincided with all tested immunoassays.

## Graphical abstract

**Figure:**
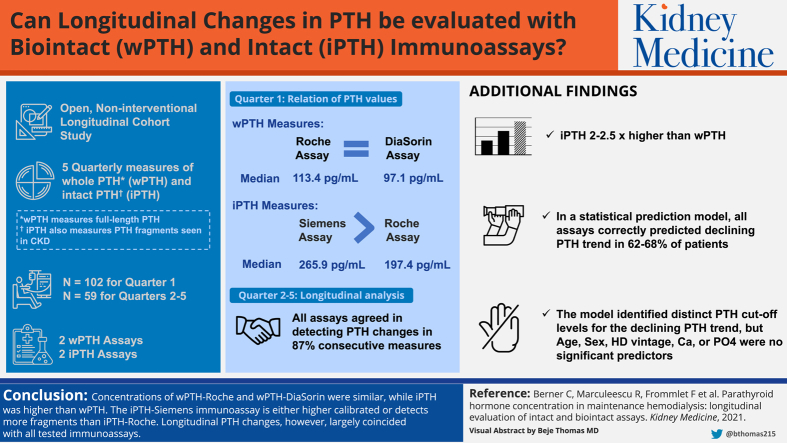

**Plain-Language Summary**

In patients with chronic kidney disease (CKD), measuring parathyroid hormone (PTH) concentration is an essential part of diagnosing and treating mineral and bone disorder. The present study was inspired by the question of whether “biointact” immunoassays that detect only full-length PTH may be required for this purpose, in view of the fact that PTH fragments accumulate in patients with CKD, and are also measured by “intact” immunoassays. We used data from 102 hemodialysis patients who underwent 5 quarterly routine examinations and studied the longitudinal test performance of 2 biointact and 2 intact immunoassays. Standard test comparisons were used, along with a prediction model for PTH, but all methods showed that the tests behaved similarly despite their distinct analytical setup.

The decline in kidney function in patients with chronic kidney disease (CKD) is associated with increasing serum phosphate, decreasing serum calcium, and increasing parathyroid hormone (PTH) concentrations.^1^^,^^2^ The KDIGO (Kidney Disease: Improving Global Outcomes) clinical practice guideline update for the diagnosis, evaluation, prevention, and treatment of CKD–mineral and bone disorder (CKD-MBD) in 2017 included the new recommendation 4.1.1, that treatment of CKD-MBD should be based on serial assessment of phosphate, calcium, and PTH concentrations, considered together.^3^ This new recommendation with an ungraded evidence level was provided to emphasize the complexity and interaction of CKD-MBD laboratory parameters.^4^

Phosphate and calcium concentrations are usually measured using colorimetric methods in automated analyzers, which portends a high degree of assay validity and a low coefficient of variation (CV).^5^ However, PTH measurements are highly variable due to diurnal variation and the presence of PTH fragments,^6^ which accumulate with decreasing kidney function,^7^ adding to an altogether low validity and high CV of PTH immunoassays.^5^ Per the previous and currently unchanged KDIGO CKD-MBD guideline 3.1.4 from 2009,^5^ the work group recommended that therapeutic decisions should be based on trends rather than on a single laboratory value. Moreover, it was stated that understanding the assay type and precision, as well as interassay variability, is required for the interpretation of biochemical and hormonal values in the diagnosis of CKD-MBD.^5^

Among various generations of PTH tests are single-site immunoassays and 2-site immunoassays for “intact” PTH (iPTH) that detect full-length (whole PTH [wPTH; 1-84 PTH) and PTH fragments.^8^ Two-site immunoassays that detect only wPTH are entitled “biointact” tests.^9^ The clinical performance of any one immunoassay is usually evaluated by a comparison of one immunoassay against another by means of correlation analyses at a single time point. Longitudinal immunoassay behavior to our knowledge has not been systematically assessed. In the current study in maintenance hemodialysis (HD) patients, we evaluated PTH, calcium, and phosphate concentrations over time, as recommended by KDIGO. Our specific aims were to: (1) provide methods for converting PTH concentrations obtained by some widely used immunoassays, (2) determine whether iPTH and wPTH immunoassays uniformly detect significant longitudinal intrapatient changes, and (3) assess using a prognostic model whether clinical factors and calcium and phosphate levels could forecast increasing versus decreasing PTH concentrations, by type of immunoassay.

## Methods

### Study Design and Participants

All patients 18 years or older undergoing uninterrupted HD or hemodiafiltration (HD patients) at our Chronic HemoDialysis (CHD) Unit in December 2017 were eligible. According to routine clinical practice, all HD patients underwent quarterly blood sample collections on December 4 and 5, 2017 (quarter 1 [Q1]); March 5 and 6, 2018 (Q2); June 4 and 5, 2018 (Q3); September 3 and 4, 2018 (Q4); and December 3 and 4, 2018 (Q5) after their 3-day interdialytic interval. Only patients who had provided written informed consent that their residual blood serum could be stored and analyzed for research purposes were included in the present study.

Patient demographics were recorded at baseline and their HD treatment characteristics were updated every quarter. These data included age, sex, height, time span receiving HD (vintage), target body weight, type of anticoagulation (heparin or citrate), treatment alterations affecting CKD-MBD (after the time points of sample collection), dialysate calcium concentration, and medications for CKD-MBD.

Approval for this open noninterventional longitudinal cohort study was obtained from the Ethics Committee of the Medical University of Vienna (EK-No. 2221/2017). The study adhered to the Declaration of Helsinki and was registered with clinicaltrials.gov (NCT03464149).

### Blood Sampling and Laboratory Analysis

Blood was drawn from the patient’s HD access after discarding at least 10 mL if patients had venous catheters to avoid contamination with catheter lock solutions. Blood was always obtained before the HD session, was allowed to clot at room temperature, and was transported to the central laboratory for analyses within 60 to 180 minutes after sampling. PTH stability could thereby reasonably be ensured.^10^^,^^11^ Routine laboratory workup included total calcium, phosphate, and creatinine (all measured from fresh sera using Cobas 8000 modular analyzer series from Roche Diagnostics), as well as iPTH and vitamin D metabolites. The latter were analyzed using immunoassays.

Aliquots were temporarily stored frozen at −80 °C in polystyrene storage tubes. PTH concentrations were measured in 1 replicate using 4 different sandwich immunoassays: (1) the Elecsys intact PTH from Roche (iPTH-R: epitopes for the monoclonal capture and detection antibodies at amino acids 26-32 and 37-42, respectively), (2) the biointact PTH immunoassay Elecsys from Roche (wPTH-R: epitopes for the monoclonal capture and detection antibodies are located at the front end of the N-terminal region including the first amino acid and at the C-terminal region, respectively), (3) the iPTH immunoassay ADVIA Centaur from Siemens (iPTH-S: epitopes for monoclonal capture and detection antibodies at amino acids 52-59 and 14-28, respectively), and (4) the biointact PTH immunoassay Liaison from DiaSorin (wPTH-D: epitopes for polyclonal capture and detection antibodies at amino acids 39-84 and amino acid 1 [=serine], respectively).

On the days of analyses, batch-wise thawing of the samples enabled PTH measurements of whole follow-up profiles using all 4 immunoassays. This procedure avoided influences of analytical day-to-day variations. Both immunoassays from Roche were run on a Cobas e602 module (Roche Diagnostics), the iPTH-S immunoassay was run on an ADVIA Centaur analyzer (Siemens Healthcare Diagnostics), and the wPTH-D immunoassay was run on a LIAISON analyzer (DiaSorin). Sample processing, calibration process, and quality control were performed according to the manufacturer’s instruction.

### Statistical Analysis

Descriptive statistics, specifically mean ± standard deviation (SD), median and interquartile range (if concentrations were not normally distributed by the D’Agostino-Pearson test^12^), and relative frequency were used to present patient baseline characteristics, treatment data, and laboratory values.

Agreements among results of both iPTH immunoassays and both wPTH immunoassays were illustrated using Bland-Altman plots.^13^ Nonparametric Passing-Bablok regressions^14^ were calculated from N=102 patients at baseline (Q1) to obtain method conversion equations among the 4 PTH immunoassays (detailed methods provided in Item S1). Linearity was confirmed using the Cusum test.^15^ Associations among results of the 4 PTH immunoassays were evaluated using Pearson correlation coefficients of log-transformed data because concentrations were skewed to the left. To obtain more insight into the reliability of PTH conversions, as calculated with the method conversion equations, we assessed concordance correlation coefficients^16^, ^17^, ^18^ of log-measured versus log-converted PTH values from a subset of N=59 patients with additional Q2 to Q5 data sets. Q1 of these patients was not used because it was a subset of the baseline PTH samples. Additionally, we calculated bias and limits of agreement^13^ (Item S1).

We also analyzed in more detail the entire longitudinal Q1 to Q5 study data from the subset of N=59 patients. We started with a data reduction of PTH concentrations obtained from the 4 immunoassays by calculating CVs and slopes for each follow-up profile. Again, we calculated Passing-Bablok comparisons, now using the reduced data.

Estimating the significance of changes is crucial in the clinical assessment of laboratory follow-up data. The reference change value (RCV)^19^ is commonly used to estimate substantial changes. Here, a simplified equation RCV = 2.8 × CV_tI_ was used, with CV_tI_ as the total individual CV across 5 consecutive PTH measurements for each patient and for each PTH immunoassay (detailed methods provided in Item S1). We computed whether consecutive concentrations significantly increased or decreased.

Because the aim of longitudinal therapy in patients with CHD is to retain the PTH concentration within a certain range, a regression-to-the-mean model^19^^,^^20^ was constructed to forecast the overall PTH trend, choosing the independent variables based on clinical considerations (Item S1). Based on clinical relevance, the following predictors (regressors) were entered into the model: age, sex, HD vintage, and serum calcium, serum phosphate, and PTH concentrations at Q1. The regression-to-the-mean model yields the predicted trend (Y) equal to a threshold minus a factor times the regressor (X). If the latter arithmetic expression is lower than the threshold, the forecasted slope is positive (ascending). Cutoff values were calculated by dividing the thresholds by the respective factors. The predictive values were calculated as the ratio of algebraic signs of real trends over predicted trends, enclosing both correct and false algebraic signs.

Data reductions, calculations, and graphs were computed using Microsoft Excel 2007, MedCalc 12.5.0.0 (info@medcalc.org), and R, version 3.6.0 (R Foundation for Statistical Computing, Vienna, Austria).

## Results

### Patient Inclusion for Cross-sectional and Longitudinal Analyses

**Figure 1:**
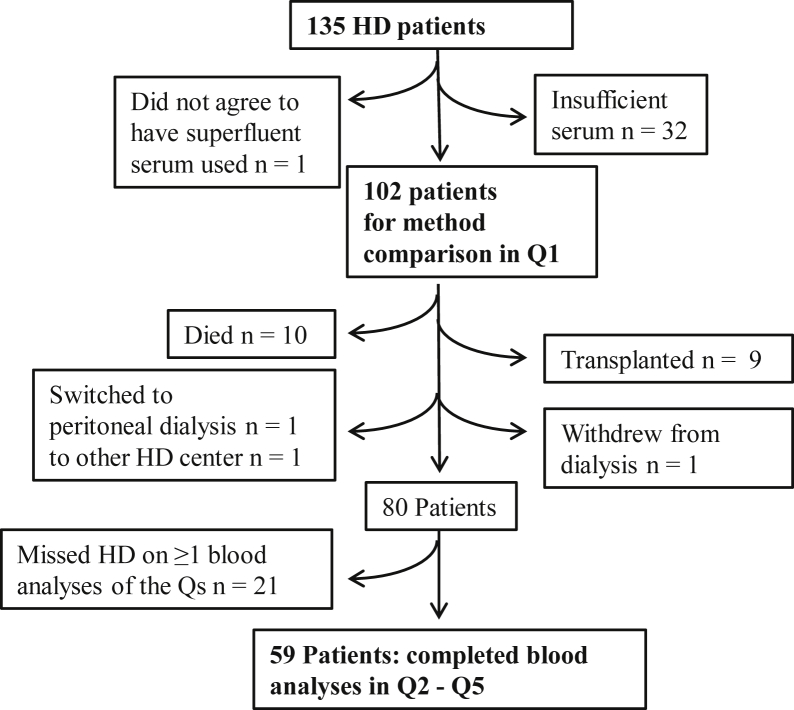
Patient flow chart. Abbreviations: HD, hemodialysis; Q, quarter.

**Figure 2:**
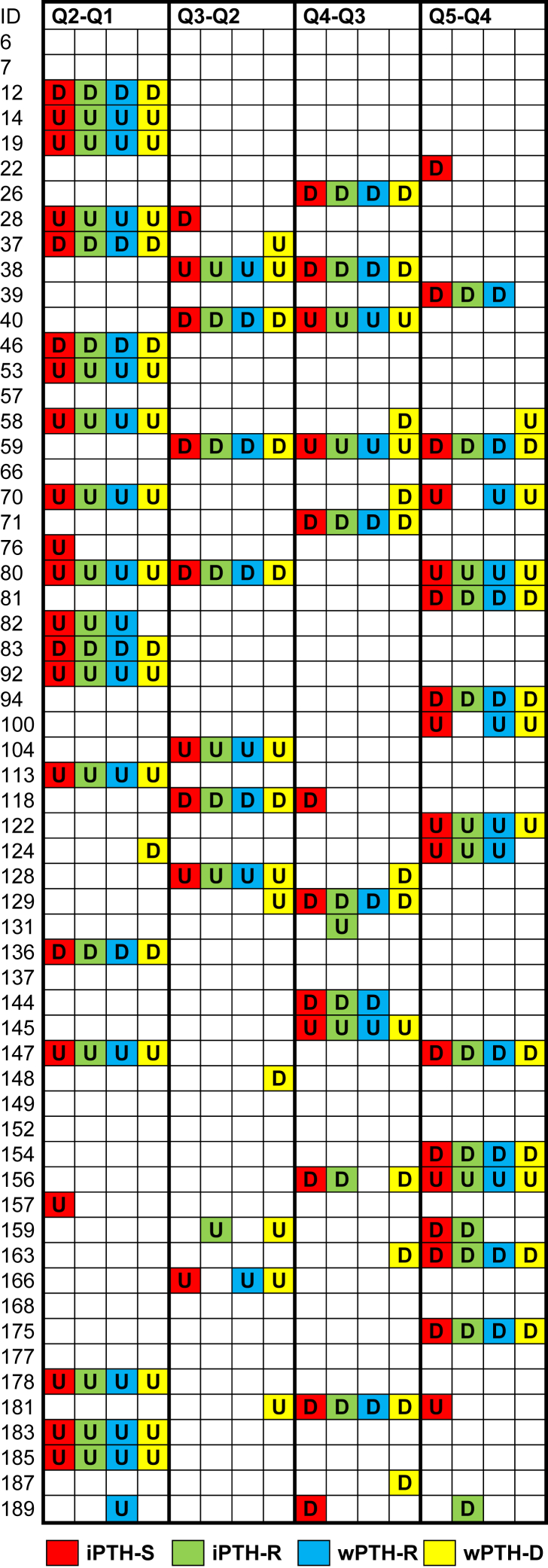
Significant changes of consecutive parathyroid hormone (PTH) concentrations as assessed by individual reference change values. Assays are encoded by different colors. Abbreviations and Definitions: D, actual value is significantly lower than the previous one; empty fields, not significant (*P*≥0.05); ID, patient identification numbers; U, actual value is significantly higher than the previous one.

Of all 135 patients dialyzing at the CHD Unit in December 2017 (Fig 1), 102 patients remained for cross-sectional evaluation at baseline (Q1; Tables 1 and 2,^21^). From Q1 forward, 59 patients did not miss any of the subsequent Q2 to Q5 blood sample collections, such that their full data could be analyzed longitudinally (Figs 1 and 2; Tables 3 and 4).

**Table 1:** Demographics, Laboratory Values, and Treatment Characteristics at Baseline (Q1)

	Total (N = 102)	Reference Range
Age, y	56.3 ± 15.7	
Female sex	51 (50.0%)	
Height, cm	168.0 ± 9.8	
Dry weight, kg	70.0 ± 17.1	
Hemodialysis vintage, mo	25.5 [9.0-49.8]	
iPTH-Siemens, pg/mL	265.9 [99.9-596.6]	18.5-88.0
iPTH-Roche, pg/mL	197.4 [76.9-432.7]	15-65
wPTH-Roche pg/mL	113.4 [53.8-230.2]	14.9-56.9
wPTH-DiaSorin, pg/mL	97.1 [36.8-222.5]	6.5-36.8
Total serum calcium, mg/dL	8.4 ± 0.8	8.8-10.6
Total serum phosphate, mg/dL	5.6 [4.6-6.5]	2.5-4.5
Serum urea nitrogen, mg/dL	65.8 ± 17.9	12-48
Serum albumin, g/L	39.4 ± 4.1	34-48
25-Hydroxyvitamin D, ng/mL	11.2 [7.2-18.8]	20-50
1,25-Dihydroxyvitamin D, pg/dL	3.8 [1.9-7.6]	2 66
Dialysate calcium concentration		
1.25 mmol/L	74 (72.6%)	
1.50 mmol/L	20 (19.6%)	
1.75 mmol/L	6 (5.8%)	
Citrate anticoagulation	2 (2.0%)	
Phosphate-binder treatment	96 (94.2%)	
Aluminum hydroxide	18 (17.7%)	
Sevelamer hydrochloride or carbonate	36 (35.3%)	
Calcium acetate	41 (40.2%)	
Calcium carbonate	1 (1.0%)	
Vitamin D agonist	60 (58.8%)	
Cholecalciferol	2 (2.0%)	
Alfacalcidol	46 (45.1%)	
Paricalcitol	5 (4.9%)	
Calcitriol	7 (6.9%)	
Calcium treatment (oral)	7 (6.9%)	
Calcimimetics	19 (18.7%)	
Cinacalcet	17 (16.7%)	
Etelcalcetide	2 (2.0%)	

*Note:* Categorical variables are reported as count and frequency. Continuous variables are reported as mean ± standard deviation or median [interquartile range], depending on their distribution. Conversion factors for units: calcium in mg/dL to mmol/L, ×0.2495; phosphate (inorganic) in mg/dL to mmol/L, ×0.3229; serum urea nitrogen in mg/dL to mmol/L, ×0,357; 25-hydroxyvitamin D in ng/mL to nmol/L, ×2.496; 1,25-dyhydroxyvitamin D in pg/mL to pmol/L, ×2.6..

Abbreviations: iPTH, intact parathyroid hormone; Q, quarter; wPTH, whole parathyroid hormone.

**Table 2:** Conversion Equations Between PTH Immunoassays

Y =	Intercept, pg/mL	+	Slope	× X	*r*^a^	*ρ*_*c*_ : Y_measured_ vs Y_calculated_ (Q2-Q5)^b^
iPTH-S =	−5.4 (−8.7 to −2.0)	+	1.33 (1.30-1.34)	× iPTH-R	0.99	0.956-0.992
iPTH-S =	−23.2 (−36.2 to −15.6)	+	2.54 (2.45-2.65)	× wPTH-R	0.98	0.969-0.981
iPTH-S =	2.8 (−6.2 to 10.3)	+	2.48 (2.36-2.67)	× wPTH-D	0.98	0.964-0.982
iPTH-R =	4.0 (1.5 to 6.5)	+	0.75 (7.40 to7.70)	× iPTH-S	0.99	0.986-0.994
iPTH-R =	−13.4 (−22.4 to −8.3)	+	1.92 (1.85-2.01)	× wPTH-R	0.99	0.954-0.986
iPTH-R =	6.7 (0.4 to 12.1)	+	1.86 (1.78-1.98)	× wPTH-D	0.98	0.963-0.974
wPTH-R =	9.1 (6.3 to 13.7)	+	0.39 (0.38-0.41)	× iPTH-S	0.98	0.968-0.980
wPTH-R =	7.0 (4.5 to 11.1)	+	0.52 (0.50-0.54)	× iPTH-R	0.99	0.974-0.987
wPTH-R =	11.7 (8.9 to 15.0)	+	0.97 (0.94-1.00)	× wPTH-D	0.99	0.967-0.975
wPTH-D =	−1.1 (−4.4 to 2.3)	+	0.40 (0.37-4.20)	× iPTH-S	0.98	0.938-0.980
wPTH-D =	−3.6 (−6.8 to 2.2)	+	0.54 (0.51-0.56)	× iPTH-R	0.98	0.955-0.970
wPTH-D =	−12.0 (−16.0 to −8.9)	+	1.03 (1.00-1.06)	× wPTH-R	0.99	0.950-0.987

*Note:* The Passing Bablok conversion equations and 95% CIs (in parentheses) were calculated from PTH concentrations at Q1 of 102 patients. When the Passing-Bablok method is applied, intercepts and slopes are calculated, based on shifted medians.^21^ Specifically, the slope is estimated by taking the shifted median of all slopes of the straight lines between any 2 points, excluding lines for which the slope is equal to 0, −1, or ±∞. Shifting the median depends on the numbers of slopes being smaller than −1. The intercept is calculated by: = median {y_i_ − *b* x_i_}. The Passing-Bablok regression analysis also uses a special method to calculate 95% CIs of intercept and slope, which help interpret the method comparison (please refer to the Supplementary Material for additional details). Note also that slope and intercept are not the midpoints of the CI calculations, according to Passing-Bablok.

Abbreviations: D, DiaSorin; iPTH, intact parathyroid hormone; PTH, parathyroid hormone; Q, quarter; R, Roche; S, Siemens; wPTH, whole parathyroid hormone.

a Pearson correlation coefficient *r* (calculated from log-transformed data; all *P* < 0.001).

b Y values were calculated from X values of N = 59 patients with each Q2 to Q5 data set using the method conversion equations. Nonsense, ie, negative Y concentrations, were omitted (they may rarely result from small X values in equations with a negative intercept). After log-transformation, measured concentrations were correlated with calculated values by using concordance correlation coefficients, and their minima and maxima from the respective Q2 to Q5 data are listed in the right column of the table.

**Table 3:** Comparison of Individual CV_tI_ From N = 59 Follow-up Profiles (Q1-Q5 measured by iPTH and wPTH assays)

CV_tI_, % Y=	Intercept		Slope	× X	Pearson *r*
iPTH-S =	2.5 (−0.3 to 4.6)	+	0.92 (0.87-0.97)	× iPTH-R	0.99
iPTH-S =	1.0 (−0.4 to 2.8)	+	0.90 (0.85-9.4)	× wPTH-R	0.95
iPTH-S =	1.4 (−1.4 to 4.2)	+	1.00 (0.94-1.07)	× wPTH-S	0.97
iPTH-R =	−1.3 (−4.0 to 0.9)	+	0.97 (0.91-1.03)	× wPTH-R	0.95
iPTH-R =	−1.2 (−4.3 to 1.1)	+	1.07 (1.00-1.16	× wPTH-D	0.96
wPTH-R =	1.7 (−0.3 to 3.7)	+	1.10 (1.03-1.17)	× wPTH-D	0.98

*Note:* Parentheses indicate 95% CIs. For simplification, only 6 immunoassay combinations are shown, by omitting the respective 6 inverted combinations. All Pearson correlation coefficients from log-transformed CV_tI_ were highly significant (all *P* < 0.001). When the Passing-Bablok method is applied, the intercepts and slopes are calculated based on shifted medians.^21^ Specifically, the slope is estimated by taking the shifted median of all slopes of the straight lines between any 2 points, excluding lines for which the slope is equal to 0, −1, or ∞. Shifting the median depends on the numbers of slopes being smaller than −1. The intercept is calculated by = median {y_i_ − *b* x_i_}. The Passing-Bablok regression analysis also uses a special, method to calculate 95% CIs of intercept and slope, which help interpret the method comparison (please refer to the Supplementary Material for additional details). Slope and intercept are not the midpoints of the CI calculations, according to Passing-Bablok.

Abbreviations: CV_tI_, total individual coefficient of variation; iPTH, intact parathyroid hormone; Q, quarter; R, Roche; S, Siemens; wPTH, whole parathyroid hormone.

**Table 4:** Summary of PTH Changes (relates to Fig 2)

	N=	iPTH-S	iPTH-R	wPTH-R	wPTH-D	Frequency
Significant changes						
4 assays	43	43	43	43	43	18.2%
3 assays	8	8	5	7	4	3.4%
2 assays	2	1	2	0	1	0.8%
1 assay	21	7	2	1	11	8.9%
Not significant	162					68.6%
Sum	236	59	52	51	59	100.0%

Abbreviations: D, DiaSorin; iPTH, intact parathyroid hormone; PTH, parathyroid hormone; R, Roche; S, Siemens; wPTH, whole parathyroid hormone.

### Patient Characteristics

Demographics and CKD-MBD–relevant treatment characteristics of our 102 study patients are shown in Table 1. Patients were on average 56 years old and median patient vintage was 2 years. Median iPTH concentrations measured with immunoassays from Siemens and Roche were nearly 3 times higher than the upper limit of the reference range of the respective immunoassay. Median wPTH concentrations measured with immunoassays from Roche and DiaSorin were 1.9 (respectively, 2.6) times higher than the upper reference limit of the respective immunoassay. Serum calcium concentrations partly overlapped with the lower reference limit, while the median serum phosphate concentration was 1.2-fold higher than the upper reference limit.

### Immunoassay Comparison at Baseline (Q1)

**Figure 3:**
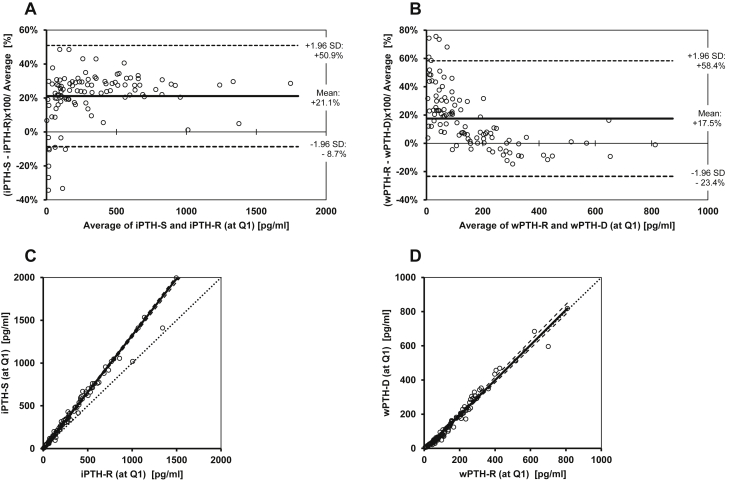
Agreement between intact parathyroid hormone [iPTH] and whole PTH [wPTH] assays. (A, B) Bland-Altman plots^13^ and (C, D) nonparametric Passing-Bablok regression plots^14^ were used to compare results of PTH concentrations of the baseline blood draw (quarter 1), measured with (A, C) iPTH-Siemens (iPTH-S) and iPTH-Roche (iPTH-R), as well as (B, D) wPTH-R and wPTH-DiaSorin (wPTH-D). (C, D) Additional information for the graphs: Passing-Bablok regression line (bold line), its 95% CIs (dashed line) and the line of identity (with slope = 1: dotted line).

For the iPTH immunoassays, the Bland-Altman percent-difference plot (Fig 3A^13^^,^^14^) revealed a mean ± 1.96 SD bias of 21.1% ± 9.8% for iPTH-S versus iPTH-R. For the wPTH immunoassays, the Bland-Altman percent-difference plot (Fig 3B) revealed a mean ± 1.96 SD bias of 17.5% ± 40.9% for wPTH-R versus wPTH-D. The latter Bland-Altman plot (Fig 3B) showed a marked concentration-dependent bias, especially below an average wPTH concentration of 300 pg/mL. Expressed in absolute concentrations, the mean ± 1.96 SD bias was 84.0 ± 180.2 pg/mL for the iPTH immunoassays (iPTH-S vs iPTH-R) and 8.2 ± 43.3 pg/mL for the wPTH immunoassays (wPTH-R vs wPTH-D), respectively.

The Passing-Bablok scatter plots, which correspond to the Bland-Altman plots, are shown in Fig 3C and D. The iPTH immunoassay from Siemens yielded higher values than the iPTH immunoassay from Roche (Fig 3C), as already derived from the positive bias shown in Fig 3A. In contrast, the comparison of both wPTH immunoassays showed a Passing-Bablok regression line close to the identity string (Fig 3D).

For any combination of iPTH and wPTH immunoassays, we observed a large mean ± 1.96 SD bias, outside of any clinical usability (absolute bias of 18%-85% and ±1.96 SD up to 53%). The results from iPTH and wPTH immunoassays therefore may only be compatible if conversion equations are applied. The Passing-Bablok regression analyses yielded the regression equations listed in Table 2, which enable method conversion calculations. Although wPTH concentrations were similar with both immunoassays, concentrations of the iPTH-S immunoassay were approximately one-third higher than concentrations of the iPTH-R immunoassay. Concentrations of the iPTH-S immunoassay were about 2.5 times higher than wPTH concentrations. Concentrations of the iPTH-R immunoassay were approximately 2 times higher than wPTH concentrations. The log(PTH) concentrations, as determined with all 4 immunoassays, were highly linearly correlated, as demonstrated by Pearson correlation coefficients, ranging between *r* = 0.98 and *r* =0.99 (all *P* < 0.001).

Because PTH concentrations measured at baseline (Q1) were selected for the method conversion equations in Table 2, we proved their validity by mutual conversions of the Q2 to Q5 results from the 4 PTH immunoassays (Table S1). All converted PTH concentrations were compared with the corresponding original PTH concentrations by concordance correlation coefficients (Table 2, right column), which are interpreted as substantial concordance^16^, ^17^, ^18^ between calculated and measured PTH values. The previous large bias between methods was thereby reduced to a few percent (Table S1).

### Longitudinal Immunoassay Comparison (Q1-Q5)

The graphical illustration of individual PTH courses over 5 quarters (December 2017 to December 2018) indicated that changes were rather parallel with all 4 immunoassays (individual data not shown). By comparing the variability, that is, the CVs from measurements with iPTH and wPTH immunoassays of 59 individual follow-up profiles, all Passing-Bablok regression lines of CV (Table 3) approximately hit the origin (intercept, −1.3% to 2.5%) and their slopes were near 1 (line of identity). Thus, the variance of the 4 PTH immunoassays was comparable.

The significance of longitudinal changes between consecutive PTH concentrations as measured by the 4 PTH immunoassays was evaluated by individual RCVs of N = 59 patients. In Fig 2, such changes were illustrated. In total, 59×4=236 consecutive values had to be evaluated for each immunoassay. Full agreement among all 4 immunoassays in detecting significant and insignificant longitudinal changes in PTH concentrations from one quarter to the next was reached in nearly 87% of consecutive measurements (Fig 2; Table 4).

### PTH Prediction Model (regression-to-the-mean model)

To gain further insight into the value and potential differences of the 4 different immunoassays, we aimed to determine predictors for PTH slopes, that is, the overall PTH concentration trend (detailed methods provided in Item S1). Irrespective of the immunoassay, only PTH concentration at Q1 was a significant predictor for PTH concentration trend (Table 5). Specifically, the PTH concentration that exceeded the calculated cutoff at Q1 was associated with a descending PTH concentration trend (a negative overall slope). However, the PTH concentration below that calculated cutoff at Q1 was associated with an ascending PTH concentration trend (a positive overall slope). The regression-to-the-mean model correctly predicted the descending and ascending trends in 64% (wPTH-R and iPTH-D), 66% (iPTH-R), and 70% of patients (wPTH-D), respectively. Thus, prognostic rates of all immunoassays were similar. None of the other laboratory or clinical variables (phosphate and calcium concentrations, age, sex, and vintage) was a significant predictor. Additionally, we failed to detect a significant correlation among either phosphate or calcium concentrations with PTH concentration, measured using the 4 immunoassays. With regard to CKD-MBD–specific therapy, very few patients received monotherapy (N = 10 with phosphate binders alone). CKD-MBD–specific therapy was therefore not entered into the model.

**Table 5:** Prediction of Overall PTH Trend

	iPTH-S	iPTH-R	wPTH-R	wPTH-D
Predicted trend = Y	Y=25.523-0.061 ×X	Y=22.397-0.058 ×X	Y=11.377-0.057 ×X	Y=12.169-0.060 ×X
*P* values of regressor = X				
PTH at Q1	<0.001	<0.001	<0.001	<0.001
Age	0.60	0.64	1.00	1.00
Sex	0.73	0.87	0.55	0.64
Vintage	0.29	0.33	0.25	0.26
Phosphate	0.30	0.30	0.32	0.40
Calcium	0.73	0.62	0.54	0.44
Cutoff, pg/mL	417.1	386.0	198.6	203.5
Ascending trend: real/predicted	63%	65%	66%	71%
Declining trend real/predicted	66%	68%	62%	67%
Ascending (declining) trend correctly predicted, N=	24 (14)	26 (13)	25 (13)	29 (12)

*Note:* The regression-to-the-mean models for the prediction of overall PTH concentration trend (slope) are based on N = 59 PTH follow-up profiles. The most important intention of this analysis was to compare the performance of the various PTH immunoassays (shown in columns 2-5). The model takes the general equation: predicted trend (Y) = intercept + slope × regressor (X). The second row shows these equations for the various assays. Rows 4-9 (*P* values of regressor = X) show whether any of the clinical variables significantly predicted the PTH trend. Row 10 shows the cutoff values for predicted trend. PTH at baseline above (below) this cutoff predicted a declining (ascending) trend. Rows 11 and 12: In about two-thirds of the predicted trends (either ascending or declining), the forecasts were correct. Row 13: The model correctly predicted the trends in 64% to 70% (sum of correctly predicted trends/all 59 trends).

Abbreviations: D, DiaSorin; iPTH, intact parathyroid hormone; PTH, parathyroid hormone; Q, quarter; R, Roche; S, Siemens; wPTH, whole parathyroid hormone.

## Discussion

From the diagnostic perspective, iPTH immunoassays detect wPTH as well as C-terminal fragments.^22^ The latter lack portions of the N-terminus, do not activate the PTH/PTH-related peptide receptor, and therefore do not mediate PTH calcemic actions such as calcium release from bone. One PTH fragment (PTH [7-84]) has been shown to antagonize the action of wPTH.^23^, ^24^, ^25^, ^26^ However, the biological significance of PTH fragments altogether remains to be defined,^22^ especially with regard to the cardiovascular system.^27^ The so-called biointact wPTH immunoassays make use of detection antibodies against epitopes at the very N-terminus and claim to measure only the biologically active full length (1-84) PTH.^9^

The following principal question underlies the present study: Does the clinician require knowledge of full-length PTH and therefore has to use the wPTH immunoassay, or can the clinician rely equally well on iPTH immunoassays, which are often less costly? The KDIGO guideline update,^3^ specifically recommendation 4.2.3, refers to iPTH levels only without explicitly recommending wPTH levels as diagnostic or therapeutic targets. The present study results suggest, in reply to this question and to the aims that were put forth, the following:

1. Method conversions: method conversions are required to gain continuity in follow-up, if a switch is desired or necessary from one PTH immunoassay to another. Even among iPTH or wPTH immunoassays, there is high bias (Fig 3). Thus, concentration results cannot be used interchangeably. Specifically, the iPTH-S immunoassay is either higher calibrated or detects more fragments than the iPTH-R immunoassay. However, despite their poor concordance, there are excellent linear correlations between concentration results from all 4 tested immunoassays (*r* = 0.98-0.99), which make method conversion equations feasible (Table 2).
2. Longitudinal PTH and significant changes: the longitudinal individual PTH variances, as expressed by CVs in a subset of 59 patients with full data through 5 quarterly checks, were similar with all 4 immunoassays (Table 3). However, the courses of the 4 measured PTH concentrations were somewhat but not perfectly parallel, indicating some longitudinal intraindividual disparity in test behavior. Besides analytical imprecision, this disparity may be explained by intraindividual differences in PTH fragment accumulation over time or by other factors influencing immunoreactivity, such as unexpected cross-reactions, for example, to oxidized PTH. These phenomena might have been caused systematically by variable degrees of residual kidney function (residual urine volume^28^) in maintenance HD patients over time, which could have led to various degrees of PTH fragment accumulation. Previous studies have shown that PTH fragment accumulation depends on the degree of kidney failure.^7^^,^^29^^,^^30^ Methodologically, our study design enabled us to evaluate significant changes during PTH courses by calculations of individual RCVs. In ∼87% of cases, all 4 immunoassays coincided in identifying significant and insignificant longitudinal changes in consecutive PTH concentrations (Table 4). Furthermore, either significant increases or decreases of PTH concentrations were consistently detected (Fig 2). The number of significant changes was slightly higher with iPTH-S and wPTH-D (Table 4), mainly because of isolated findings, not confirmed by other immunoassays. This result can be interpreted as higher diagnostic sensitivity or as reduced specificity because significant changes in a longitudinal setting have more emphasis if detected by several immunoassays.
3. Predictive model: our regression-to-the-mean model identified only baseline PTH (PTH at Q1) as a significant predictor for a long-term PTH concentration trend, independent of the type of immunoassay (Table 5). Baseline PTH cutoff levels correctly forecasted the direction of the 1-year PTH concentration trend (ascending or declining) in up to 70% of cases.

Among this study’s limitations, we acknowledge that our results are derived from only 2 iPTH and wPTH immunoassays, respectively. Thus, the interchangeability that we observed concerns only these PTH immunoassays and tests from other manufacturers may behave differently. Furthermore, only 102 patients from a single maintenance HD center were available for cross-sectional evaluation at baseline, and the subset of patients with full data through Q5 was even smaller. Although the validation of method conversion equations was performed with the PTH results of this subset, only tests after baseline were used, known to have some interassay disparity. We are aware that in our previous cross-sectional analysis on wPTH-R, wPTH-D, and iPTH-R,^9^ we arrived at Passing-Bablok regression equations that slightly differed from those that we identified in the present study. The 95% CIs of previous and current intercepts coincided, but not the 95% CIs of the slopes. There are at least 8 years between these measurements on different patients by different generations of Roche analyzers and different reagent lots. In the meantime, Roche Diagnostics changed the concentrations in their calibration set but did not change the standardization and, to date, the reagent composition. However, we cannot expect that long-term calibration is totally stable. Moreover, the intraindividual disparity of biointact/intact PTH ratios may play an even greater role, as emphasized by the absence of perfect agreement in the Passing-Bablok regression equations among the various quarters.

As additional limitations, the RCVs, evaluated retrospectively and individually, were applied only on neighboring quarterly checks but not on half-year or 1-year intervals. The prediction model should not be used to rank the immunoassays because some trends were very flat and the number of observations is too small for this endeavor. However, that only baseline PTH concentration was a predictor for PTH slope (ie, descending PTH trend above a baseline PTH cutoff) may deserve confirmation in additional data sets.

The strengths of this study include its centralized laboratory analysis and thorough analysis of the method conversions. To our knowledge, our study is the first to systematically examine the longitudinal course of PTH concentrations, which is recommended by KDIGO,^3^ and we did so by using various immunoassays. Because the iPTH-S is new on the market, the present study is also the first to evaluate its clinical performance.

In conclusion, the results from all tested PTH immunoassays yielded comparable information about the course of PTH concentrations in maintenance HD patients. Although PTH concentrations varied systematically between the iPTH and wPTH immunoassays, as expected by their different binding capacities to PTH fragments, the examined immunoassays are to a large extent convertible among one another. Significant (and insignificant) changes were mostly detected in consonance of iPTH and wPTH immunoassays. In a predictive model, baseline PTH concentration was a predictor for PTH slope, independent of the type of immunoassay. Translating these results into clinical usability, in which an understanding of PTH trends is required for the treatment of CKD-MBD, each of the 4 immunoassays that was tested provides reliable longitudinal information that can inform and/or guide treatment decisions. We believe that this study’s message on the longitudinal comparability of PTH courses, which was previously expected but never proven, is an important facilitation for clinical practice.

## Supplementary Material

Supplementary File (PDF) Figure S1, Item S1, Table S1.
