# Supplementary material for: Parathyroid Hormone Concentrations in Maintenance Hemodialysis: Longitudinal Evaluation of Intact and Biointact Assays
Source: Kidney Med. 2021 Feb 27;3(3):343–352.e1. doi: 10.1016/j.xkme.2020.12.015 (PMC8178455; doi:10.1016/j.xkme.2020.12.015)
Supplement: Supplementary File (PDF) — Figure S1, Item S1, Table S1. [file mmc1.pdf]

Item S1. Short explanations of methods used in this study

Bland-Altman Plot[13] is a graphical procedure to test agreement of methods, but it is sensitive to outliers. The differences of paired values obtained with two methods (Y-axis) are plotted against their average (X-axis). In %-difference plots the differences are related to the corresponding averages. The mean difference ± 1.96 SD represents the bias and the limits of agreement of the methods. If differences within these limits are clinically not important, the two methods may be used interchangeably.

Passing–Bablok regression[14] is a non-parametric regression analysis suitable for method comparison studies with at least 50 paired observations and a linear relationship between the methods X and Y. This symmetrical procedure (suitable for X vs. Y or Y vs. X) is robust in the presence of few outliers and fits the parameters *a* and *b* of the linear equation $y = a + b x$. The slope *b* is estimated by taking the shifted median of all slopes of the straight lines between any two points, excluding lines for which $b = 0, -1 \text{ or } \pm \infty$. Shifting of the median depends on the numbers of slopes being $b < -1$. The intercept *a* is calculated by $a = \text{median } \{y_i - b\, x_i\}$. The Passing-Bablok regression analysis uses special methods to calculate 95% confidence intervals (CI) of *a* and *b*,[30] which help to interpret the method comparison: If "0" is not within the CI of *a*, then there is a systematic difference and if "1" is not within the CI of *b*, then there is a proportional difference between the two methods that are being compared. Note that intercept and slope are not the midpoints of the calculated CIs (please compare **Figure 2 C, D**). Passing-Bablok equations can be used for method conversions, if the correlation between the methods is significant.

Concordance Correlation Coefficient[16-18] = Peason's correlation coefficient × bias-factor

$\rho_c = (r \times f_{bias})$

Pearson's correlation coefficient is valid for normal distributions. Values skewed to the left have to be log-transformed to approach a normal distribution (see below "Method conversion").

Reference Change Value (RCV)[19] to assess significance of differences for serial results is widely used, e.g. in the follow-up of cancer patients by tumor markers to predict progression, remission and stable disease or by bone resorption or formation markers to evaluate bone turnover. To be significant, the absolute difference in serial results must be greater than the combined variation inherent in the two results x_i and x_{i+1}: abs $|x_{i+1} - x_i| / x_i$ > RCV.

The formula $RCV = 2^{1/2} \times Z \times (CV_A^2 + CV_I^2)^{1/2}$ takes the statistics ($2^{1/2} \times$ Z-score) of the biological variation as well as of the analytical variation into account. CV_A: analytical coefficient of variation (e.g. from quality control data of analytic imprecision); CV_I: within subject biological variation (e.g. from estimates in the literature); Z-score: number of standard deviations appropriate to the probability (e.g. http://www.z-table.com/t-value-table.html). If subsequent results do not differ significantly, a rather stable concentration course is assumed.

PTH may either increase (U[p] = $x_{i+1} > x_i$) or decrease (D[own] = $x_{i+1} < x_i$), therefore we used the two-sided Z = 1.96 at a confidence level of 95%. The total individual CVs, as calculated from the N=59 longitudinal PTH measurements include both, the analytic and individual variability across 5 consecutive observations per patient $CV_{tI} = (CV_A^2 + CV_I^2)^{1/2}$, replacing the Pythagorean operation to combine CV_A and CV_I. Thus, the

simplified equation $RCV = 2.8 \times CV_{tI}$ was used to assess the significance. Afterwards we evaluated the direction of PTH change.

Regression-to-the-mean model:[20] Regression lines were fitted to the longitudinal measurements of each of the four PTH immunoassays from every of the 59 patients with complete data. The resulting slopes were used as outcome variables for linear regression models. Slopes from each PTH immunoassay were analyzed separately.

First a simple regression model only including the baseline value of PTH was fitted, and the baseline value was always significant. Then this model was augmented by age, sex, hemodialysis vintage, phosphate and calcium, respectively. None of these additional regressors was significant for any of the four immunoassays. Based on the simple regression model considering the baseline value of PTH as a predictor of the slope we estimated for each immunoassay the cut-off point where the slope is expected to become negative when baseline values become larger than this cut-off point.

Method conversion is a tool in medical laboratories, if a switch in methods is either desired or necessary, but the agreement of results is low due to proportional and/or systemic differences, despite good correlation. Such conversions rest upon e.g. Passing-Bablok equations and are important especially in longitudinal observations to convert previous results for connecting them with those of the new method. However, the limitations are random variability in methods and low values to be converted. Variability in immunoassay results may be caused by analytic imprecision or by immunologic disparity. Low concentrations together with a negative intercept (systemic deviation) may

produce nonsense i.e. negative values. On the other hand low values together with low slope (proportional deviation) may be converted to outlier in % difference plots.*

After mutual conversions of the Q2 to Q5 results from the four PTH immunoassays and omitting negative values, we tested for concordance between original and converted PTH concentrations by calculating the mean % difference ± 1.96 SD. We arbitrarily expected to reduce the bias to at least ± 10 % with still remaining, assay-inherent limits of agreement of up to about ± 50%. The Bland-Altman plots systematically revealed outliers at low PTH concentrations which would influence the interpretation, a typical example is shown in **Figure S1A**. To eliminate these outliers we empirically tried thresholds of 5, 10 and 20 pg/ml for the averages plotted on the X-axis. Eliminating the averages over measured and converted concentrations < 10 pg/ml was best balanced between removing outliers and not too much data (see example in **Figure S1B**). Percental bias and agreement limits are summarized in **Table S1**. Only 8 out of 48 combinations marginally missed our proposed targets. Assessment from cross-sectional data of method conversions will give a bias of only few percent with agreement levels of about ±35%. Nevertheless, there is a substantial concordance between measured and converted concentrations (**Table 2**)

* Examples:
Conversion equation: iPTH-S = -23.2 + 2.54 wPTH-R
e.g. iPTH-S measured = 6.6, wPTH-R measured = 5.5 → iPTH-S(calculated from wPTH-R) = -9.2

Conversion equation: wPTH-D = -1.1 + 0.4 iPTH-S
e.g. wPTH-D measured = 4.0, iPTH-S measured = 4.0 → wPTH-D(calculated from iPTH-S) = 0.5
difference of measured-calculated = 3.5, average of measured and calculated = 2.3
% difference = 156%

Figure S1 Examples of Bland-Altman Plots: Rejection of outliers

A

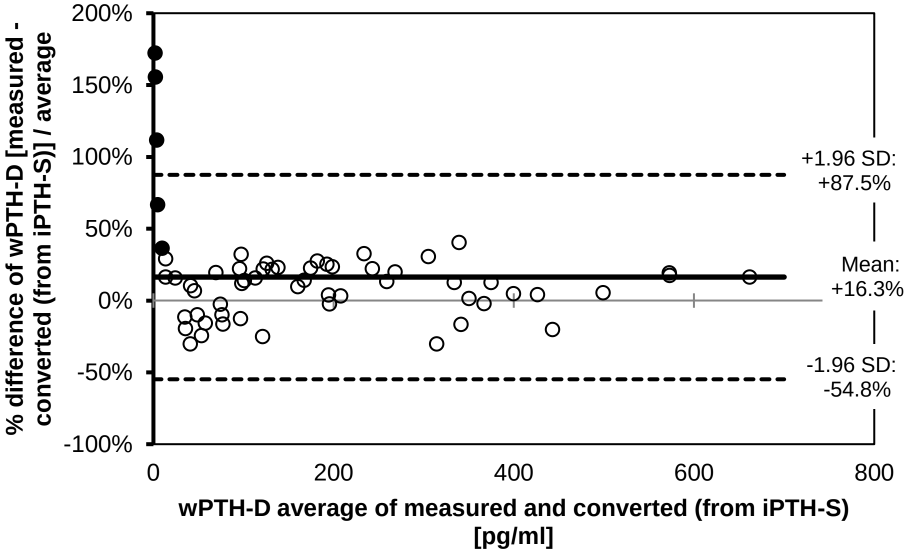

B

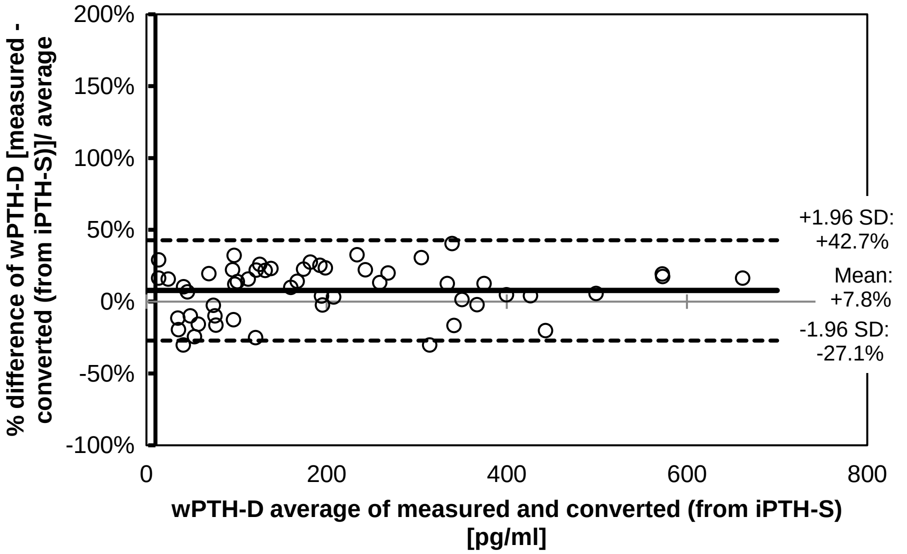

Legend for Figure S1:

Data were used from Q5

A: Range of X-axis 0 to maximal, •…average <10 pg/ml, data points defined as “outliers”

B: Range of X-axis 10 to maximal, “outliers” removed

Table S1: Conformity between measured and converted PTH according to percental bias ± limit of agreement

**Conversion equations**						**Bias ± 1.96 SD [%] (n=)**					
**Y =**	**intercept [ng/L]**	**+**	**Slope**	**x**	**X**	**Q2**	**Q3**	**Q4**	**Q5**	**Average Bias [%]**	**Average 1.96 SD[%]**
iPTH-S =	-5,4	+	1,33	x	iPTH-R	-3,0 ± 17,5 (57)	-2,6 ± 26,4 (58)	-10,3 ± 26,1 (57)	-10,0 ± 35,4 (56)	-6,5	26,3
iPTH-S =	-23,2	+	2,54	x	wPTH-R	-2,8 ± 34,8 (57)	-0,8 ± 32,6 (56)	-10,0 ± 37,4 (56)	-8,1 ± 38,1 (55)	-5,4	35,7
iPTH-S =	2,8	+	2,48	x	wPTH-D	7,4 ± 39,5 (58)	-7,6 ± 39,9 (59)	-7,8 ± 42,5 (57)	-9,6 ± 41,5 (57)	-4,4	40,8
iPTH-R =	4,0	+	0,75	x	iPTH-S	3,2 ± 16,8 (57)	2,8 ± 24,2 (59)	10,4 ± 25,0 (57)	10,4 ± 33,5 (56)	6,7	24,9
iPTH-R =	-13,4	+	1,92	x	wPTH-R	-0,4 ± 28,2 (57)	2,8 ± 33,1 (56)	0,1 ± 37,0 (56)	1,4 ± 33,1 (55)	1,0	32,9
iPTH-R =	6,7	+	1,86	x	wPTH-D	9,9 ± 39,1 (58)	-4,5 ± 48,0 (59)	1,0 ± 51,6 (58)	0,8 ± 51,0 (57)	1,8	47,4
wPTH-R =	9,1	+	0,39	x	iPTH-S	3,5 ± 29,7 (57)	-0,6 ± 38,8 (58)	10,2 ± 32,7 (56)	7,9 ± 35,4 (56)	5,2	34,2
wPTH-R =	7,0	+	0,52	x	iPTH-R	0,6 ± 25,9 (57)	-3,2 ± 32,2 (57)	0,2 ± 33,8 (56)	-1,3 ± 30,0 (55)	-0,9	30,5
wPTH-R =	11,7	+	0,97	x	wPTH-D	7,3 ± 35,4 (58)	-8,2 ± 38,2 (58)	-1,6 ± 42,6 (58)	-3,8 ± 47,4 (58)	-1,6	40,9
wPTH-D =	-1,1	+	0,4	x	iPTH-S	-7,4 ± 39,1 (57)	6,3 ± 38,0 (55)	8,1 ± 42,6 (56)	7,8 ± 34,9 (54)	3,7	38,7
wPTH-D =	-3,6	+	0,54	x	iPTH-R	-11,9 ± 39,1 (56)	0,5 ± 42,7 (55)	-3,9 ± 48,1 (56)	-3,6 ± 50,3 (54)	-4,7	45,0
wPTH-D =	-12,0	+	1,03	x	wPTH-R	-8,2 ± 49,6 (57)	6,4 ± 30,8 (56)	-2,8 ± 22,7 (55)	-3,0 ± 23,7 (53)	-1,9	31,7

The Passing-Bablok regression equations[14] computed from PTH of 102 patients at Q1 served to convert X_{PTH} (as measured from 59 patients at each Q2- to Q5- check) to Y_{PTH}. Nonsense results, i.e. negative Y_{PTH} concentrations were omitted from calculations as well as averages of measured and converted PTH <10 pg/ml, to overcome outliers. The percental bias ± 1.96 SD was calculated according

to Bland-Altman[13] from %(measured PTH –converted PTH) ×100/average PTH. The actual number of paired observations is shown in brackets (N=).
